# Why Do Patients Seek Diagnose Dis-accordance With Hierarchical Medical System Related Policies in Tertiary Hospitals? A Qualitative Study in Shanghai From the Perspective of Physicians

**DOI:** 10.3389/fpubh.2022.841196

**Published:** 2022-03-25

**Authors:** Yuhui Ruan, Jin Luo, Hong Lin

**Affiliations:** ^1^School of Politics and Public Administration, Soochow University, Suzhou, China; ^2^Institute of Public Health, Soochow University, Suzhou, China; ^3^School of International and Public Affairs, Shanghai Jiao Tong University, Shanghai, China; ^4^Institute of Health Yangtze River Delta, Shanghai Jiao Tong University, Shanghai, China; ^5^Department of Orthopaedic Surgery, Zhongshan Hospital, Fudan University, Shanghai, China; ^6^Institute of Medical Science Popularization, Fudan University, Shanghai, China

**Keywords:** diagnose-seeking behavior, tertiary hospitals, Hierarchical Medical System, reasons, qualitative study, grounded theory

## Abstract

**Background:**

Although the Hierarchical Medical System has been utilized in China for many years, it is inadequate for guiding patients in adopting appropriate diagnose-seeking behaviors in accordance with related policies. This study examined how patients' diagnose-seeking behavior in tertiary hospitals that is dis-accordance with Hierarchical Medical System related policy (“DSB-dis-accordance”) arise and ways to guide patients away from such behaviors, both from the perspective of physicians.

**Methods:**

A qualitative study based on a mixed method including in-depth interviews and grounded theory. Twenty-seven physicians with more than 2 years of experience serving in tertiary hospitals of Shanghai were involved after reviewing the related purposes and requirements. Patients' “DSB-dis-accordance” was studied from the perspective of physicians.

**Results:**

Patient-related factors (habits, trust, and knowledge), physician-related factors (conservative preference, risk avoidance), and system-related factors (accessibility, operability) affected patients' diagnose-seeking behavior.

**Conclusions:**

Patient-related, physician-related, and system-related factors affecting patients' diagnose-seeking behaviors in tertiary hospitals should be addressed by investing more health resources in lower-level hospitals, enhancing dissemination of health-related and policy-related knowledge, refining the classification of diseases, incentivizing physicians, and developing appropriate follow-up measures. Physicians could then become more involved in guiding patients' “DSB-dis-accordance,” thereby benefitting development of the Hierarchical Medical System in China.

## Background

The Hierarchical Medical System has been employed in China for many years to optimize the allocation of medical resources and relieve the burden on the medical system (1–3). The guidelines suggest that patients with common, frequently occurring, and/or chronic diseases should first seek diagnosis at primary medical institutions. Patients with acute, severe, or difficult-to-treat diseases can be transferred to higher-level institutions, including secondary and tertiary medical institutions, for further treatment. However, the effects of this system are far from ideal. Many patients continue to seek diagnosis contrary to these guidelines. Many patients with acute, severe, or difficult-to-treat diseases seek diagnosis at tertiary hospitals (referred to as diagnose-seeking behavior in tertiary hospitals that is dis-accordance with Hierarchical Medical System related policy in this study, “DSB-dis-accordance” for short). The system does not seem to effectively guide patients to seek medical care at lower-level hospitals (4, 5). To overcome this problem, increasing attention has focused on studying how related policies guide patients' diagnose-seeking behaviors. Patients' diagnose-seeking behavior is the result of a complex decision-making process involving multiple aspects that can be affected not only by the patients themselves but also physicians and the social health context (6). However, the results of research efforts to date have not been effective at guiding patients to adopt appropriate diagnose-seeking behaviors in accordance with related policies. Few studies have examined how physicians impact patients' diagnose-seeking behaviors. Thus, to develop a solution to guide patients' “DSB-dis-accordance,” this qualitative study was conducted from the perspective of physicians in Shanghai, China.

### Development of Hierarchical Medical System in China

In 2009, the Chinese government announced that it will establish Universal Health Coverage in China to provide safe, effective, convenient and affordable health services to all Chinese people by 2020 (7). In the same year, the Hierarchical Medical System was launched in China to guide patients seeking health services to the appropriate corresponding hospitals (8). According to related policies, medical institutions in China are divided into three levels: first-level medical institutions provide preventive, medical, health, and rehabilitation services; secondary medical institutions provide comprehensive medical and health services; and tertiary medical institutions provide high-level specialized medical and health services. To support the implementation of this policy, there is a clear policy bias in reimbursement rates. Although the reimbursement rates are inconsistent across different types of insurance, including Urban Employee Basic Medical Insurance, Basic Medical Insurance, and New Rural Cooperative Medical Scheme, it is common that in lower-level hospitals can get a higher reimbursement rate. The objective of this hierarchy is to ensure that limited medical resources are allocated efficiently and economically. As reported previously, however, this otherwise well-designed system has exhibited limited effectiveness (9). Lower-level hospitals are less competitive than higher-lever—particularly tertiary—hospitals (10). Relatively few patients seek care according to the system's design (11, 12), resulting in “DSB-dis-accordance.” For example, many patients with diseases or conditions that would be more appropriately diagnosed at lower-level medical institutions still insist on seeking diagnosis at tertiary hospitals. As a result, medical care becomes increasingly unaffordable for many patients and the model's sustainability in terms of providing health care, especially at higher-level institutions, is significantly challenged (13).

### DSB-Dis-accordance and Related Factors

A patient's diagnose-seeking behavior reflects their decision-making, which in turn is affected by many personal, psychosocial, and cultural factors (14). It also depends on a patient's ability to acquire medical resources, including perceiving, seeking, reaching, paying, and engaging (15). In addition, social context and physical environment have a significant effect on personal health behavior (16, 17). Activities pertaining to involvement, social norms, certainty, and perceived availability are associated with patients' health behavior as well (18).

Researchers focusing on the related topic also reported that motivation and pressure associated with rejecting reforms affect personal behavior to a different degree (19). Patient attitudes (20), together attitudes of others also can influence an individual's diagnose-seeking behavior (21). Some studies even suggest that individuals will react according to the system recommendations only when they conclude that they will benefit from referrals made by doctors (22).

### Knowledge, Trust, Accessibility, Physicians and Patient's Diagnose-Seeking Behaviors

Additionally, patients' knowledge and trust, accessibility, and suggestions from their physicians can guide their diagnose-seeking behaviors (6, 23–25).

#### Knowledge

Health-related knowledge can provide patients with needed theoretical methods to elicit health-seeking behavior change (23, 26). Limited knowledge is found associated with patient's inappropriate first-diagnosed seeking behavior (27). Thus, public health policy makers, health practitioners and educators are focusing on enhancing individual's knowledge by health education to change their behaviors (28), like smoking (29), nutrition (30), practice toward epidemic (31), and so on. But studies about related association with patient's health-seeking behavior is still limited, especially lacking study from the perspective of physicians.

#### Trust

Patient's trust in the physicians (32), medical institutions (17), and chronic conditions (33), are found to be important for their health. Related trust can enhance patients' adherence to institutions and physicians (34), and even impact on the health results positively (35). Thus, many studies are focusing on enhancing individual's trust on related health sources (36), treatment (37), public health policies (38), by education. Increasing researchers believe patients' higher trust in their health care treatment can result in beneficial health behaviors (39). Thus, it is meaningful to study the association of trust with patient's diagnose-seeking behaviors.

#### Accessibility

Accessibility is highly associated with usability (40). Accessibility, including opportunity to seek healthcare services, to reach, to obtain or use health care services (15), can impact the performance of health care system greatly. Health resources accessibility has challenged modern city planning and population health (41). Thus, related accessibility is regarded as important social determinants of health (42). It just that the association of patient's diagnose-seeking behavior and related accessibility is still in development. Thus, more work needs to be done.

#### Physicians

Physicians play important roles in affecting patients' health behaviors. First, delivery of high-quality care is a well-known responsibility of physicians (i.e., the “patient-centered” approach) (43, 44). Guiding patients' diagnose-seeking behaviors to promote their health equity is always also a physician's responsibility, and this is important for maintaining the physician-patient relationship (45). Second, the knowledge gap between what the patients are seeking and what the physicians are providing has been widened by rapid technical developments (46). The professional medical knowledge of physicians has enlarged their influence on patients' diagnose-seeking behaviors (47). Despite knowledge limitations, it is the responsibility of physicians to assess patients' risks in treatment processes and understand the efficacy of related treatments (48). Therefore, the influence of physicians on patients' diagnose-seeking behaviors is obvious. However, although the responsibilities and professional influence of physicians are widely acknowledged, many practicing physicians cannot fully understand them in related processes (49). Meanwhile, lack of trust between patients and physicians is another challenge (50).

The current Hierarchical Medical System faces clear challenges associated with patients' “DSB-dis-accordance.” It is therefore essential to determine what motivates patients related diagnose-seeking behaviors from the perspective of physicians and consequently determine how to redirect them from the perspective of physicians. This qualitative study was designed to address these questions.

## Methods

### Study Setting

**Figure 1:**
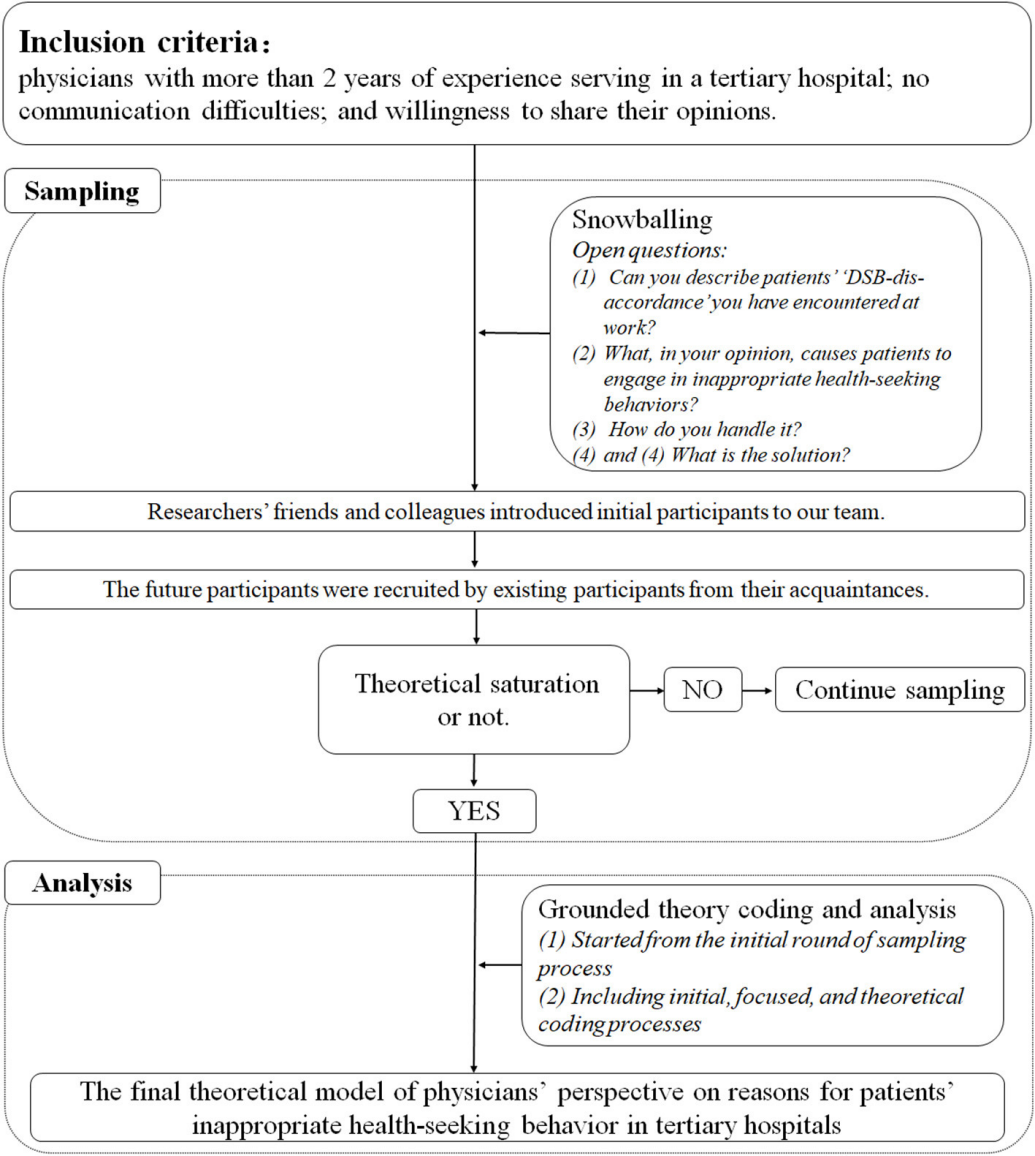
Study design.

A qualitative study based on a mixed method of in-depth interviews and grounded theory was conducted. Participants were recruited via a snowballing process. With open questions, coding, comparing, memo writing, and immediate data analysis, the study proceeded until data saturation. The final theoretical model of reasons leading to patients' “DSB-dis-accordance” from the perspective of physicians emerged and was interpreted (Figure 1).

Grounded theory is a systematic methodology for developing theories (51, 52). As a study progresses, a theory will emerge and gradually become clear. Grounded theory emphasizes inductive analysis and provides an advantage relative to normative approaches in developing new theories or hypotheses. Related data collection is more interactive. It is adopted and supposed to enrich the data of in-depth interviews. In this study, grounded theory facilitated development of a theoretical model for exploring “DSB-dis-accordance” from the perspective of physicians and informed efforts to devise ways to guide related behaviors. Although the number of patients engaging in related behavior is vast, relatively little attention has been focused on how physicians impact patients' diagnose-seeking behaviors. Theoretical results are quite limited, to the best of our knowledge. As grounded theory carries the advantage of assisting researchers in developing theories, it can help fill in gaps in current research.

### Sampling

#### Inclusion Criteria

Most participants were unfamiliar with the researchers before enrollment in this study. Participants were first briefed on the method and aims of the study. The inclusion criteria for the participants were as follows: physicians with more than 2 years of experience serving in a tertiary hospital (from all departments were included/targeted); no communication difficulties; and willingness to share their opinions.

#### Sampling Process

All participants were recruited from tertiary hospitals of Shanghai *via* a snowballing process. Researchers' friends and colleagues introduced initial participants to our team. All participates were interviewed by the two corresponding authors of this study to ensure consistency. Both of these two researchers were trained in in-depth interview/qualitative data collection. After confirming the potential participants met the inclusion criteria of this study, the researchers explained the purpose and the process of the study to them. After confirming the participates fully understood related contents and was willing to involve in this study (verbal informed consent), the time and manner of in-depth interview was negotiated. Two types of in-depth interview manners were offered in this study, namely, online and offline. The online manner required each participant and the researchers to conduct the in-depth interview in a separate space, *via* webcam. The offline manner was conducted by the researchers and the participants together in a separate space free from distractions. Each in-depth interview last for 40–60 mins. The researchers transcribed the progress during the interview and the voice was recorded simultaneously. After the in-depth interviews were completed, all participants were encouraged to recommend additional participants who met the inclusion criteria to join the study. They were promised the right to be informed of the progress of this study. Meanwhile, coding, comparing, memo writing, and immediate data analysis were continuously carried out. One researcher transcribed the recorded data into textual material based on the field notes. The other proofread and reviewed it. For some uncertain content they discuss with each other and even the participates to ensure the accuracy of the information collected. When the first round of data was coded, potential participants for the second round were screened based on recommendations from this round of participates and more others. A second round of sampling was then conducted as above. Similarly, the third round of sampling was conducted, until data saturation.

The study was designed to examine “DSB-dis-accordance” from the perspective of physicians (differences in patients' and physicians' opinions in understanding and reasons for related behaviors were not the focus of this study). Thus, participants in this study were required to provide their opinions about “DSB-dis-accordance” based on their professional judgment regarding common, frequently occurring, and chronic diseases.

All individuals were recruited from July to December 2019. Anonymized references (both letters and numbers) were adopted in this study to ensure confidentiality.

This study was approved by the School of International and Public Affairs, Shanghai Jiao Tong University. All procedures followed were in accordance with the ethical standards of the responsible committee on human experimentation (institutional and national) and with the Helsinki Declaration of 1975, as revised in 2000 (5). Informed consent was obtained from all patients for being included in the study.

### Data Collection

In-depth interview was the primary data collection method used in this study. This approach has been widely employed in qualitative studies based on grounded theory (53, 54). In-depth interviews can help researchers more clearly describe the meanings of themes central to the lives of the participants (55), and was therefore employed in this study.

The initial open questions for the in-depth interview used in this study were as follows: *(1) Can you describe patients' “DSB-dis-accordance” you have encountered at work? (2) What, in your opinion, causes patients to engage in patients' related behaviors? (3) How do you handle it?* and *(4) What is the solution?* The primary reasons for patients engaging in “DSB-dis-accordance” from the perspective of physicians involved three themes, as determined in the second round of sampling and coding: “patient-related,” “physician-related,” and “context-related.” Reasons falling into these themes were considered to play an essential role in leading patients to engage in related behaviors in this study. Therefore, these reasons were examined in a more-focused manner in the third round of sampling and in-depth interviews. Finally, the study explored how “DSB-dis-accordance” occur from the perspective of physicians and how these behaviors should be guided.

### Data Analysis

Theoretical sampling and independent data coding analysis were carried out simultaneously by the corresponding authors in this study. All data were analyzed in a cumulative manner. Group discussions were held regularly to exchange information and communicate ideas in time since the beginning. With the analysis went on, special discussions were held to determine the themes. Special experts in the field were consulted to handle some encountered disagreements. “*What is the relationship between various factors and patients' diagnose-seeking behavior in tertiary hospitals that is dis-accordance with Hierarchical Medical System related policy?”* emerged as the open-up question in this study. Initially, there was no answer to this question. This study focused on developing a theoretical model via subsequent rounds of sampling and analysis. In the later stages of sampling and theoretical coding, by using the constant comparative method (comparing codes against codes and data against data), three themes were identified, as described above. A theoretical model describing the three aspects of patients' “DSB-dis-accordance” (patient-related, physician-related, and context-related) and their relationship with various factors was developed.

## Results

**Figure 2:**
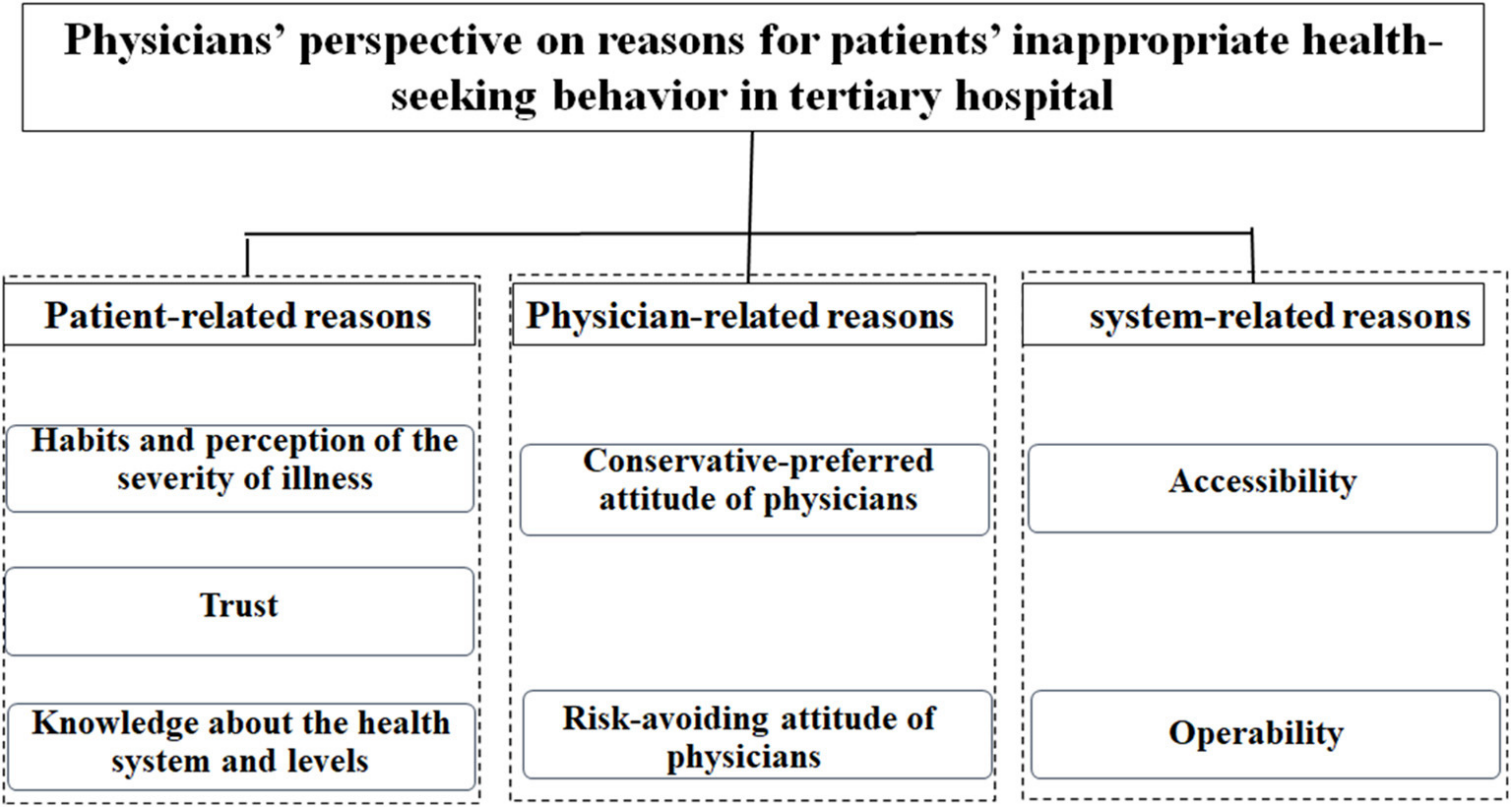
Results identified by the qualitative study.

A total of 27 physicians with more than 2 years of experience serving in tertiary hospitals of Shanghai, China (tertiary hospitals involved here includes Zhongshan Hospital of Fudan University, Huashan Hospital of Fudan University, Eye, Ear, Nose and Throat Hospital of Fudan University, Jinshan Hospital of Fudan University, Ruijin Hospital of Shanghai Jiao Tong University, Renji Hospital of Shanghai Jiao Tong University, Ninth People's Hospital of Shanghai Jiao Tong University, Sixth People's Hospital of Shanghai Jiao Tong University) were enrolled after explanation of the related purposes and requirements (Table 1). The study examined patients' “DSB-dis-accordance” from the perspective of these physicians (Figure 2).

**Table 1:** Basic participant information.

**No**.	**Gender**	**Age**	**Education**	**Service length**	**Department**
NX-1	Male	41	Doctor	7 years	Urology surgery
LJ-2	Male	40	Doctor	5 years	Orthopedics
YHSN-3	Male	39	Doctor	5 years	General surgery
CHX-4	Female	37	Doctor	5 years	General surgery
TJ-5	Male	40	Doctor	8 years	Orthopedics
LFZ-6	Male	39	Doctor	3 years	Orthopedics
LLH-7	Male	36	Doctor	4 years	General surgery
ZXB-8	Male	34	Doctor	2 years	Infections
GW-9	Female	33	Doctor	2 years	Cardiology
WF-10	Male	35	Doctor	3 years	Sports medicine
GLS-11	Male	38	Doctor	5 years	General surgery
LY-12	Male	43	Doctor	10 years	Neurology
ZHR-13	Male	37	Doctor	4 years	Sports medicine
LC-14	Female	38	Doctor	5 years	Dermatology
ZP-15	Male	36	Doctor	4 years	Sports medicine
FC-16	Male	37	Doctor	5 years	Gastroenterology
JMH-17	Female	32	Doctor	3 years	Urology surgery
ZCF-18	Male	32	Master	3 years	General surgery
HZJ-19	Male	46	Master	5 years	Rehabilitation medicine
LMH-20	Male	37	Master	5 years	Integrated traditional and Western medicine
ZYH-21	Male	53	Bachelor	15 years	Urology surgery
YU-22	Female	46	Master	8 years	Gastroenterology
OL-23	Male	43	Doctor	6 years	Traditional Chinese medicine
PSD-24	Female	33	Doctor	3 years	Plastic surgery
SWM-25	Male	36	Doctor	7 years	Urology surgery
ZTJ-26	Male	44	Doctor	6 years	General surgery
SSJ-27	Male	39	Doctor	4 years	Plastic surgery

### Patient-Related Reasons for Inappropriate Diagnose-Seeking Behaviors

#### Habits and Perception of the Severity of Illness

Patients' diagnose-seeking behavior were generally based on their past experience and habits. Many patients have long held the opinion that tertiary hospitals are the best choice for optimizing their health outcomes. Such habitual thinking led to “DSB-dis-accordance” for these patients.

*This is actually quite tricky. Because many patients just chose a higher-level hospital habitually by considering such “DSB-dis-accordance” can improve their health outcome more. The real problem is about their habitual thinking. (ZXB-8)*.

Many patients habitually chose a higher-level hospital due to improvements in the standard of living, accumulated wealth, and perspective on the value of life. Because of this habitual preference, higher-level hospitals experienced higher health service demands. Thus, many patients' diagnose-seeking behaviors are less associated with the requirements of related policies and more associated with habitual personal choices. Habits were thus considered a patient-related reason for their “DSB-dis-accordance” from the perspective of the physicians.

“*DSB-dis-accordance” are often influenced by their habitual preferences. From the patients' perspective, they must go to a tertiary hospital to see a doctor to meet their diagnose-seeking demands. (TJ-5)*.

#### Trust

In the opinion of most participants, patients generally exhibited distrust of regional/primary hospitals. In comparison, they exhibited greater trust in tertiary hospitals, including the medical staff, medical resources, and management standards, and whether related behaviors were appropriate or not did not factor into the decision to seek health services there.

*I don't believe the patients considered whether their diagnose-seeking behavior in tertiary hospitals accordance to related policies or not. They trust their own consideration more. (LJ-2)*.

The consensus was that compared to secondary hospitals or fundamental health institutions, tertiary hospitals offered the best health resources. The best line-up of experts converge at tertiary hospitals, and teams of specialized physicians had access to advanced medical equipment and drugs. All of these considerations formed the basis of patient trust in tertiary hospitals. Thus, trust was considered another patient-related reason for their “DSB-dis-accordance.”

*Obviously, the patients trust the tertiary hospitals more. In their opinions, a higher level often means a better service. Patients naturally want to trust a better hospital, which is looked more professional, with more advanced equipment. (GLS-11)*.

In contrast, primary hospitals have a long history of having an insufficient supply of medical devices. Even if a few devices are available, they are often old. The number of primary care physicians at these hospitals is often limited as well, and the quality of care they provide is also lower compared with that in tertiary hospitals. Additionally, information asymmetry can exacerbate distrust between patients and physicians. Although patients might have received adequate health care in lower-level hospitals in the past, they often are unsatisfied with the outcomes. These patients therefore shift their trust to tertiary hospitals in hopes of obtaining better care.

*So many patients believe that they must go to a higher-level hospital (i.e., a tertiary hospital) with higher-quality medical resources. These patients generally believe that tertiary hospitals can provide a wider array of drugs. Drugs prescribed in tertiary hospitals are not always available at local hospitals. (LMH-20)*.

The trust many patients place on tertiary hospitals is also affected by advertisements that focus on tertiary hospitals and overlook lower-level local hospitals. Thus, that high-quality health resources are more available at tertiary hospitals is widely known, which further increases patient trust in tertiary hospitals. Compared to tertiary hospitals, secondary and community hospitals tend to be restricted and not trustworthy. Thus, many patients seek diagnosis from the more-trusted tertiary hospitals without considering whether their diagnose-seeking behaviors are accordance with related policy or not.

*Advertisements often focus on tertiary hospitals, about how good they are, what breakthroughs they have achieved, etc. By contrast, little is advertised about primary and secondary hospitals. Such bias lowers patient trust in primary and secondary hospitals. Thus, they often seek diagnosis at trusted tertiary hospitals. (LC-14)*.

### Knowledge About the Health System and Levels

This study found, in the opinion of many physicians, that patients generally exhibited very limited understanding about health and diseases based on their limited biomedical knowledge. Additionally, many physicians also pointed out that patients' limited policy-related knowledge impacted their diagnose-seeking behavior. Both of these issues were considered patients' knowledge-related reasons in this study.

*Actually, many patient's “DSB-dis-accordance” are the results of limited knowledge, both biomedical and political. You know, it is contradictory that many patients are required to behave accordance with Hierarchical Medical System related policy without mastering related knowledge which should be the prerequisite. (JMH-17)*.

Without specialized knowledge, patients were not able to determine their state of health and therefore knew little about which level of hospital was appropriate. Thus, the prerequisite that patients abide by the Hierarchical Medical System based on the classification of diseases was not met. This was considered by many physicians to be an important factor affecting patients' “DSB-dis-accordance.”

*Patients didn't fully understand their disease based on their limited biomedical knowledge. If you want them to seek health care appropriately, you must ensure that they know what is happening with their health. How can they choose correctly without understanding what has happened? They certainly will go to the best hospital. (YU-22)*.

The Hierarchical Medical System and corresponding policy are not yet broadly known by the public. Most patients cannot grasp the procedure. Thus, related policy can play a limited role in appropriately guiding patients' diagnose-seeking behaviors. To avoid additional trouble and secure their rights by receiving better health care, it is common for many patients to seek diagnosis with little regard for related policy.

*Patients are generally unfamiliar with the process of seeking diagnosis accordance. The most important thing for patients is ensuring their health. With regard to the Hierarchical Medical System, most people don't understand it, let alone follow it. (GW-9)*.

### Physician-Related Reasons

#### Conservative-Preferred Attitude of Physicians

As a group of specialists who have been working on the front lines for a long time, most participants in this study had encountered a large number of “DSB-dis-accordance.” They also knew they were responsible for guiding such behaviors. In practice, however, all interviewed physicians claimed that upon encountering related diagnose-seeking behaviors, they provided the health services requested by patients universally, doing their best to provide such services as complete health checks, decent treatment, and so on.

*It is common to encounter* “DSB-dis-accordance.” *I know I am responsible for guiding such behaviors if I am free. But I am not free. I always just provide them with necessary health service directly. Most patients prefer to be treated right away to get rid of their health troubles than to know where to seek diagnose is more appropriate. It is less acceptable for physicians to educate them at this moment about things that are not related. (YU-22)*.

Generally, most physicians believed providing health services to meet patients' needs was their ethical responsibility as a health care provider. Meanwhile, most patients with related diagnose-seeking behavior had non-serious medical issues that were easier to handle. It was easier and less troublesome for physicians to provide patients with health care services than guide them toward diagnose-seeking behavior that was accordance with Hierarchical Medical System related policy.

*After registration, the symptoms patients have usually fall within our specialty. They came from far away to seek health services from physicians here, and we couldn't turn them away right at the moment. For example, although a foot sprain is simple to treat, we would usually provide the patient with an appropriate examination as well. It is easier and less troublesome. (ZP-15)*.

Thus, physicians' attitudes toward guiding related behaviors were conservative. Physicians preferred not to intervene in patients' diagnose-seeking behaviors. This was considered as a “conservative-preferred' attitude, which had minimal impact on changing patients” diagnose-seeking behavior that was dis-accordance with Hierarchical Medical System related policy.

#### Risk-Avoiding Attitude of Physicians

Most physicians were “risk-avoiding” when encountering patients' “DSB-dis-accordance.” As the participants reported, it was difficult to point out related dis-accordance and guide them appropriately, which might place their work at potential risk.

*You have to know that we all love our patients. But we all never want to get in trouble. It was a risk for us to point out related dis-accordance and guide them appropriately. We are easy to be considered as discriminate them, the consequences of which will be serious. (LLH-7)*.

It was clear that most physicians were risk-avoiding when facing potential physician-patient conflicts. The physicians knew that the law requires them to provide patients with appropriate health services. By contrast, intervening in patients' diagnose-seeking behaviors was not strongly supported by policies. In the physicians' opinions, disagreement between a physician and patient was an important issue. Guiding patients' diagnose-seeking behaviors might increase risk and cause more misunderstanding between them and their patients. Thus, most physicians exhibited little confidence in pointing out patients' “DSB-dis-accordance” and had little incentive to do so. Most considered it “pointless” to do so.

*We don't usually point out patients' “DSB-dis-accordance.” In my opinion, refusing to take patients or pointing them out are pointless. It does not help develop a good physician-patient relationship. In contrast, if the patients misunderstand why and think we're rejecting them, there will be more trouble. I believe every physician wants to avoid such risk. (WF-10)*.

Additionally, most participants believed that behavioral interventions to guide patients diagnosis-seeking accordance with Hierarchical Medical System related policy carried the risk of increasing their workload. Taking the intensity of their job into account, many physicians were too busy. They were not willing to take the risk and devote more time to trivial matters that had little to do with their medical diagnosis. Thus, they generally intervened very little.

*We don't point out their accordance with diagnose-seeking behaviors generally. We service so many patients every day and don't have time to explain why it's dis-accordance. Any carelessness in this process may cause unnecessary trouble for us. It's more likely to cause risk, such as physician-patient conflicts, and extra workload. (LMH-19)*.

### System-Related Reasons

#### Accessibility

In the opinion of many physicians, most diagnose-seeking behavior that was dis-accordance with Hierarchical Medical System related policy resulted from various system-related factors. One of the most frequently mentioned reasons was increased accessibility to quality health care resources.

*There are about 6–7 communities around our hospital. It is easy for them to access the quality health care resources here. Why don't they come? (LFZ-6)*.

Generally, most tertiary hospitals are located in the most populated city centers and surrounded by many neighborhoods. Nearby residents prefer to seek health services in these tertiary hospitals no matter whether this behavior is accordance with related policy or not. With the presence of highly developed transportation systems, even residents who do not reside nearby have convenient access to these quality health resources as well. In addition, advanced facilities and management and online registration platforms have greatly increased access to tertiary hospitals. Such accessibility was considered a reason for patients' “DSB-dis-accordance.”

*Take Shanghai as an example, where tertiary hospitals are so dense. Meanwhile, its public transportation system is very well developed. The online platforms are also well done. If there were no regulations restricting or manipulating their behaviors, many patients would not go to ordinary hospitals. (ZTJ-26)*.

#### Operability

Another reason physicians believe the Hierarchical Medical System related policy is ineffective relates to lack of operability. The policy encourages patients to take advantage of primary and secondary hospitals. However, it has set no specific binding procedures to guide patients' diagnose-seeking behaviors.

*The patients are not experts. No of them are educated or trained. Policies requires them to behave appropriate without teach them how to operate. Therefore, many of them behaved dis-accordance with related policies. (SSJ-27)*.

That is to say, patients lack operational standards in relevant processes. In addition, there are no incentives for physicians to guide patients' diagnose-seeking behaviors. Consequently, the system fails to regulate the physicians' responsibility to direct patients to appropriate hospitals. Moreover, in order to effectively guide patients' diagnose-seeking behaviors, related policies must encourage the optimal allocation of health resources between hospitals to enable collaboration. However, the applicable policy is weak and lacks operability. Hospitals thus generally do not follow the policy. As a result, patients' “DSB-dis-accordance” receive minimal guidance due to a lack of operability.

*Most people are ignorant. They can't be guided by accurate information of related policy to seek health service appropriately. When people are sick, they scramble to make a choice. Without enough accurate information, they choose tertiary hospitals by instinct. The reality is either the hospital is public or private, skillful or unprofessional, and they all compete for the same market. Collaboration among hospitals regarding use of medical resources is rare. This game rule makes tertiary hospitals win all the prizes in the market. Swarming crowds are the result rather than cause of orderless competition. (ZTJ-26)*.

## Discussion

This study found that patients' “DSB-dis-accordance” is driven by patient-related reasons (habits, trust, and knowledge), physician-related reasons (conservative-preferred, risk-avoiding), and system-related reasons (accessibility, operability). Patients' diagnose-seeking preferences, along with lack of physician enthusiasm for behavior guidance, have already been reported as reasons motivating diagnose-seeking behavior that was dis-accordance with Hierarchical Medical System related policy (56, 57). Our study validates these conclusions. Thus, to guide changes in patient behavior in terms of seeking health care in accordance with the Hierarchical Medical System related policy, a supportive context that addresses all of the above-mentioned reasons is needed. Accordingly, future strategies should focus more on guiding “DSB-dis-accordance,” which could benefit further development of the Hierarchical Medical System in China.

Personal characteristics of patients, including habits, trust, and knowledge, were revealed as important drivers of patients' “DSB-dis-accordance.” First, habits have a significant impact on patient diagnosis-seeking choices (58, 59). Second, long-held negative stereotypes of lower-level hospitals and information asymmetry between patients and physicians can lead to patient distrust of lower-level hospitals. Third, patients generally have limited knowledge of health and health-related policies, but the popularization of related knowledge plays a decisive role in directing patient behavior (23, 26). As revealed in this study, many patients habitually seek routine diagnosis at tertiary hospitals. They tend to trust tertiary hospitals more than lower-level hospitals and thus seek diagnosis in tertiary hospitals. Generally, tertiary hospitals receive more subsidized medical resources than lower-level hospitals; this gives them the opportunity to advertise and build a reputation among patients. Such advantages have a tremendous effect on patients' altitudes toward hospitals and their diagnose-seeking behaviors (21, 60). As a result, patients prefer to go to tertiary hospitals and rarely change their diagnose-seeking behaviors according to referral policies. Meanwhile, most patients find referral policies too complex and therefore difficult to understand, which erodes support for policies and impedes their implementation. Information technology only further directs patients to crowd into tertiary hospitals. For these reasons, many patients prefer tertiary hospitals and go to these hospitals in masse. To enhance the current system's ability to appropriately guide patients' diagnose-seeking behaviors, much attention must be paid to changing their behavioral habits, long history of trust in tertiary hospitals, and their limited knowledge of the system and policies. This means more publicity should be given to exemplary diagnose-seeking behaviors that are accordance with related policy. In addition, positive reports about primary care hospitals should be further strengthened, and more energy should be directed toward enhancing the popularity of health sciences among patients. By investing more health care resources in lower-level hospitals, enhancing public knowledge of health-related and policy-related issues, and screening clinics and patient counseling facilities at the tertiary care hospitals, patients' “DSB-dis-accordance” might be re-directed.

Physician-related factors are also associated with patients' “DSB-dis-accordance.” There are always many different views about whether and how physicians should guide patients' diagnose-seeking behaviors. On one hand, the demands of physician education in China have their origins in complexities. “Health First” and “guiding patients' behavior” in an appropriate manner involve both career and ethical responsibilities (61). On the other hand, conflicts between physicians and patients have long existed (62). Burnout among physicians is widely reported as being the result of adverse social contexts, including high requirements about extrinsic efforts and over-commitment, low personal decision authority, supervisor support, and skill discretion (63). Such unfriendly social contexts have increased considerable pressure for physicians to guide patients' diagnose-seeking behaviors. Thus, when faced with patients' “DSB-dis-accordance,” physicians appeared to be conservative-preferred and risk-avoiding in this study. From the patient perspective, they can choose the best option for medical treatment for themselves. Obtaining health services is always central to patients' diagnose-seeking behaviors, as it markedly affects their health-related behaviors (64). The choice a patient makes might be underpinned by socioeconomic and cultural values. Thus, as revealed, they always tend to seek and obtain needed health services in tertiary hospitals, irrespective of the legitimacy of their behaviors. Physician's indiscriminate attitude toward helping patients has no effect on related policy dis-accordance diagnose-seeking behaviors. In addition, due to a lack of systematic support, many physicians have become used to avoiding pointing out and intervening in patients' diagnose-seeking behaviors after providing them with services. Most physicians have little confidence in behavioral interventions because they believe the outcome will be undesirable. Due to this lack of intervention by physicians, it is difficult to change patients' diagnose-seeking behavior. Therefore, to enhance the current system's ability to guide patients' diagnose-seeking behaviors, considerable attention should be paid to refining the classification of diseases. It will be necessary to develop more policy approaches that incentivize physicians by clarifying their responsibilities and rights with regard to providing health services and intervening in patients' “DSB-dis-accordance.” More energy should be directed toward reducing the burden of physicians, so that they have the energy to guide patients' diagnose-seeking behaviors. Meanwhile, more publicity should be given to exemplary appropriate physician-patient relationship. Then the physicians' conservative-preferred and risk-avoiding attitudes can be improved, and more of them will want to guide patients' policy dis-accordance diagnose-seeking behaviors.

System-related factors, including accessibility and operability, were also found in this study to be important drivers of patients' “DSB-dis-accordance.” Without clear guidance, patients' policy dis-accordance diagnose-seeking behaviors are supported by convenient access to high-quality medical resources in tertiary hospitals. Public transportation, residence location, and information technology lead large numbers of patients to crowd tertiary hospitals. Also, system-related issues with policies of the Hierarchical Medical System also cause difficulties for patients and physicians. Operability is important for the function of the health system and its related policies. The operability of a health-related policy should be demonstrated at various levels. Additionally, relevant training and exercises for affected populations, modification of follow-up measures, and mechanisms to promote even more collaboration are needed (65). Therefore, to guide patient's diagnose-seeking behaviors, optimizing the operability of related policies is an urgent need. More attention should be paid to increasing the accessibility of lower-level hospitals and increase their quality.

To implication of finding for policy and practice, public health educators need to enhance public knowledge of health-related and policy-related issues, and popularity of health sciences. Public health policy makers should pay more attention to refining the classification of diseases, optimizing the operability of related policies, and developing more policy approaches to incentive physicians. The governments should invest more in lower-level hospitals, increase their accessibility and quality, give more publicity to exemplary appropriate physician-patient relationship, especially that in primary care hospitals.

Although this study has some strengths, it also has several limitations. First, the snowball sampling list was derived from the participates who were initially involved, which might result in some similarity between samples. As a result, the sample may not be a good representation of the overall population. Second, participants were recruited from tertiary hospitals in Shanghai, without screening physicians from special departments. Therefore, the limited sample size and sampling method limit the generalizability of the findings to a more extend scope. Meanwhile, some departments are only set in higher-level hospitals, which should be considered specially in the further study. Third, possible self-selection bias could have led to inappropriate conclusions if the participants tried to emphasize particular positive or negative opinions or if some physicians focused on their own interests and refused to express particular views. This is a general limitation associated with our qualitative methodology. Also, with regard to this limitation, although “resource allocation” might be an important reason patients engage in “DSB-dis-accordance,” more argumentative processes are needed to verify this possibility. Finally, to identify methods to appropriately guide patients' diagnose-seeking behaviors, a study conducted from both the patients' and physicians' perspectives is needed. However, the present study was designed to examine the problem only from the perspective of physicians; it was therefore too limited to consider the perspective of patients. Thus, additional studies with an expanded sample size also conducted from the perspective of patients are needed. However, these limitations do not abrogate the insights generated by the study with regard to clarifying the difficulties associated with guiding patients' “DSB-dis-accordance.”

## Conclusions

Patients' “DSB-dis-accordance” were studied from the perspective of physicians *via* a qualitative study based on a mixed method of in-depth interviews and grounded theory. The study found that patient-related (habits, trust, and knowledge), physician-related (conservative-preferred, risk-avoiding), and system-related (accessibility, operability) factors affect patients' diagnose-seeking behaviors in tertiary hospitals. These factors should be addressed by investing more health resources in lower-level hospitals, enhancing public understanding of health-related and policy-related information, refining the classification of diseases, incentivizing physicians, and developing follow-up measures. Physicians can then become involved in positively guiding patients' diagnose-seeking behaviors.

## Data Availability Statement

The raw data supporting the conclusions of this article will be made available by the authors, without undue reservation.

## Ethics Statement

The studies involving human participants were reviewed and approved by Ethics Committee of Shanghai Jiao Tong University. The patients/participants provided their written informed consent to participate in this study.

## Author Contributions

YR and JL contributed conception and design of the project and data collection. YR and HL wrote the first draft of the manuscript. All authors contributed to data analysis, wrote sections of the manuscript, contributed to manuscript revision, read, and approved the submitted version.

## Conflict of Interest

The authors declare that the research was conducted in the absence of any commercial or financial relationships that could be construed as a potential conflict of interest.

## Publisher's Note

All claims expressed in this article are solely those of the authors and do not necessarily represent those of their affiliated organizations, or those of the publisher, the editors and the reviewers. Any product that may be evaluated in this article, or claim that may be made by its manufacturer, is not guaranteed or endorsed by the publisher.
